# Editorial Note: Malaria in Dielmo, a Senegal village: Is its elimination possible after seven years of implementation of long-lasting insecticide-treated nets?

**DOI:** 10.1371/journal.pone.0352472

**Published:** 2026-06-29

**Authors:** The PLOS One Editors

The *PLOS One* Editors issue this notice to update the previously published Expression of Concern on this article [1,2].

Following the publication of the article and Expression of Concern [1,2], PLOS investigated concerns pertaining to the reported ethical approval and the article’s adherence to *PLOS One’s* research ethics policies.

The Materials and Methods section in [1] reports that the study involved the use of blood samples, as well as daily home-based surveillance, monitoring of all episodes of fever, and repeated cross-sectional surveys involving home visits by technicians and questionnaires about the use of long-lasting insecticide-treated nets. The article reports that the study ran between August 2007 and July 2015. In addition, the study used the human landing catch method to catch mosquitos for the entomological study. The article reports that written informed consent was obtained from all participants or their legal guardians, and that the study was approved by the Ministry of Health of Senegal, the assembled village population and the National Ethics Committee of Senegal. The article does not report an ethics approval reference number.

A representative of the Aix-Marseille Université Ethics Committee stated that at the time the initial study was implemented, the National Ethics Committee for Health Research in Senegal had not yet been established, and that the ethics approvals for the earlier part of the study were instead granted by the Senegalese Ministry of Health. The representative stated that the village of Dielmo is part of a major longitudinal research site for malaria epidemiology, that the research conducted in this village is part of a continuous, closely monitored program implemented in collaboration with national health authorities and the local population, and that the documents listed below confirm that the research program was conducted in compliance with recognized standards and in accordance with good research practices under continuous ethical oversight. They provided the documents N° 969 MSPM/DS/DER, N° 1971 MSPM/DS/DER, N° 2167 MSP/DS/DER, N° 00.86 MSP/DS/CNERS, N° 001380 MSP/DS/DER, and N° 00081 MSAS/DGS/DS/CNERS for editorial review:

- Document N° 969 MSPM/DS/DER was issued on September 25, 2006 by the Ministère de la Santé et de la Prévention Médicale of the République du Sénégal, and provides ethics approval for a study titled, *“Etude de l’histoire naturelle du paludisme: protocole d’étude mené dans les villages de DIELMO et NDIOP arrondissement de Toubacouta Région de Fatick/SENEGAL”.*
- Document N° 1971 MSPM/DS/DER was issued on September 29, 2006, by the Ministère de la Santé et de la Prévention Médicale of the République du Sénégal, and provides approval for a study titled “*Etude multicentrique des facteurs immuno-génétiques impliqués dans la protection contre le paludisme*”
- Document N° 2167 MSP/DS/DER was issued on September 30, 2008, by the Ministère de la Santé et de la Prévention of the République du Sénégal. The document does not appear to be an ethics approval document, and instead it appears to be an acknowledgement of receipt of a letter from the Steering Committee of the Dielmo and Ndiop project.
- Document N° 00.86 MSP/DS/CNERS was issued on June 02, 2010, by the Ministère de la Santé et de la Prévention of the République de Sénégal and provides ethics approval for a study titled “*Identification des agents pathogènes responsibles de fièvre au Sénégal. Réalisation de tests diagnostiques chez les malades consultant dans les dispensaires de Dielmo et Ndiop*”.
- Document N° 001380 MSP/DS/DER was issued on May 31, 2011, by the by the Ministère de la Santé et de la Prévention of the République de Sénégal, and provides a one-year administrative authorization for the protocols SEN 21/09 and SEN 37/09 related to a study titled *“Identification des agents pathogènes responsibles de fièvre au Sénégal*. *Réalisation de tests diagnostiques chez les malades consultant dans les dispensaires de Dielmo, Ndiop, Niakhar, Mlomp, Bandafassi et Keur Momar Sarr.”*
- Document N° 00081 MSAS/DGS/DS/CNERS was issued on June 04, 2012, by the Ministère de la Santé et de l’Action Sociale of the République de Sénégal and provides ethics approval for protocols SEN 21/09 and SEN 37/09 related to a study titled *“Identification des agents pathogènes responsibles de fièvre au Sénégal*. *Réalisation de tests diagnostiques chez les malades consultant dans les dispensaires de Dielmo, Ndiop, Niakhar, Mlomp, Bandafassi et Keur Momar Sarr.”*

PLOS reviewed the documentation provided by the institution and concluded that the documents did not fully resolve the journal’s concerns. Specifically, it is unclear whether the documentation provided covers the full study period reported in the article, and the titles of the studies listed in the approval documents are not a clear match with the study described in [1]. The ethics approval documents do not provide much detail of the study approved by the document beyond the titles, such that it remains unclear to PLOS whether the approval for the study described in [1] was covered by these documents. In light of the unresolved issues, the Expression of Concern stands.
